# Comparison of Diaphragmatic Stretch Technique and Manual Diaphragm Release Technique on Diaphragmatic Excursion in Chronic Obstructive Pulmonary Disease: A Randomized Crossover Trial

**DOI:** 10.1155/2019/6364376

**Published:** 2019-01-03

**Authors:** Aishwarya Nair, Gopala Krishna Alaparthi, Shyam Krishnan, Santhosh Rai, R. Anand, Vishak Acharya, Preetam Acharya

**Affiliations:** ^1^Department of Physiotherapy, Kasturba Medical College, Manipal Academy of Higher Education, Bejai, Mangalore-575004, India; ^2^Department of Radiodiagnosis, Kasturba Medical College Mangalore, Manipal Academy of Higher Education, Mangalore-575004, India; ^3^Department of Pulmonary Medicine, Kasturba Medical College, Manipal Academy of Higher Education, Mangalore-575004, India

## Abstract

**Background:**

Chronic Obstructive Pulmonary Disease (COPD) impairs the function of the diaphragm by placing it at a mechanical disadvantage, shortening its operating length and changing the mechanical linkage between its various parts. This makes the diaphragm's contraction less effective in raising and expanding the lower rib cage, thereby increasing the work of breathing and reducing the functional capacity.

**Aim of the Study:**

To compare the effects of diaphragmatic stretch and manual diaphragm release technique on diaphragmatic excursion in patients with COPD.

**Materials and Methods:**

This randomised crossover trial included 20 clinically stable patients with mild and moderate COPD classified according to the GOLD criteria. The patients were allocated to group A or group B by block randomization done by primary investigator. The information about the technique was concealed in a sealed opaque envelope and revealed to the patients only after allocation of groups. After taking the demographic data and baseline values of the outcome measures (diaphragm mobility by ultrasonography performed by an experienced radiologist and chest expansion by inch tape performed by the therapist), group A subjects underwent the diaphragmatic stretch technique and the group B subjects underwent the manual diaphragm release technique. Both the interventions were performed in 2 sets of 10 deep breaths with 1-minute interval between the sets. The two outcome variables were recorded immediately after the intervention. A wash-out period of 3 hours was maintained to neutralize the effect of given intervention. Later the patients of group A and group B were crossed over to the other group.

**Results:**

In the diaphragmatic stretch technique, there was a statistically significant improvement in the diaphragmatic excursion before and after the treatment. On the right side, p=0.00 and p=0.003 in the midclavicular line and midaxillary line. On the left side, p=0.004 and p=0.312 in the midclavicular and midaxillary line. In manual diaphragm release technique, there was a statistically significant improvement before and after the treatment. On the right side, p=0.000 and p=0.000 in the midclavicular line and midaxillary line. On the left side, p=0.002 and p=0.000 in the midclavicular line and midaxillary line. There was no statistically significant difference in diaphragmatic excursion in the comparison of the postintervention values of both techniques.

**Conclusion:**

The diaphragmatic stretch technique and manual diaphragm release technique can be safely recommended for patients with clinically stable COPD to improve diaphragmatic excursion.

## 1. Introduction

Chronic Obstructive Pulmonary Disease (COPD) is a common preventable and treatable disease that is usually progressive, characterized by persistent airflow limitation and associated with an enhanced chronic inflammatory response in the airways and the lung to noxious particles or gases [1]. In India, chronic respiratory diseases account for 3% of Disability Adjusted Life Years (DALYs); together COPD, asthma, and other respiratory diseases are the second leading cause of death in the Indian population and fourth in the world according to WHO [2, 3].

The diaphragm, which is the main inspiratory muscle, generates a craniocaudal movement of its dome during contraction [4]. The two striking features in COPD, air trapping and lung hyperinflation, impair the function of the diaphragm, shortening its operating length and changing the mechanical linkage between its various parts thereby placing it at mechanical disadvantage [5]. These pathological changes affect the diaphragm's ability of raising and expanding the lower rib cage which may lead to a decrease in the transverse diameter of the lower ribcage during inspiration. These changes cause an increase in the work of breathing and reduce the functional capacity [6, 7]. Skeletal muscle dysfunction, which is an observed extrapulmonary effect in COPD, affects the severity of the disease, providing a potential target for therapeutic intervention [1]. This dysfunction occurs at a level which affects exercise capacity and dyspnoea levels [8].

Although the main treatment approaches for COPD are pharmacological therapy and pulmonary rehabilitation, there are a number of published studies in the osteopathic and chiropractic literature describing the use of manual therapy techniques [9–12].

Some evidence suggests that manual therapy has the potential to affect and change respiratory mechanics in certain chronic pulmonary diseases, such as chronic asthma and COPD, which includes an increase in flexibility of the chest wall and thoracic excursion. This can indirectly lead to an improvement in exercise capacity and lung function [13, 14]. There is also evidence that respiratory muscle stretching may bring about an improvement in ventilation in patients with COPD by increasing the capacity for chest wall (CW) expansion [15, 16].

According to some studies, stretching of the respiratory muscles improves vital capacity, improves chest wall mobility, and reduces dyspnoea thereby counteracting the effects of COPD [17]. Studies also suggest that a vast variety of manual techniques targets the various components of pulmonary system like musculoskeletal, lymphatic, etc. Studies support that manual therapy of the diaphragm improves its excursion thereby improving respiratory mechanics, facilitating bronchial tree lymphatic flow and reducing airways congestion [18–21].

The diaphragmatic stretch technique or doming of diaphragm technique is designed to relax the resting state of the diaphragm, enhancing its contraction and relaxation functions, thereby creating a greater pressure gradient between the thorax and abdomen [22].

González-Álvarez FJ et al. applied the diaphragm stretch technique to check ribcage and abdominal excursion in healthy subjects and found a significant increase in the same level at xiphoid level [23]. Yelvar YDG studied the immediate effects of manual therapy on inspiratory muscle strength and respiratory functions in patients who were a known case of COPD with no current or ongoing exacerbation, by applying the Redoming of diaphragm technique which showed an improvement in pulmonary function and inspiratory muscle strength [24].

The Manual Diaphragm Release Technique (MDRT) is an intervention intended to directly stretch the diaphragmatic muscle fibres thereby increasing the chest wall mobility [25, 26]. Evidence supports the beneficial effect on diaphragmatic mobility and it can be hypothesised that there is traction of the lower rib cage in a cranial direction and manual compression of the tissues in the area of insertion of the anterior costal diaphragm fibres brought about by the manual action on the underside of the last four costal cartilages which allows lengthening the diaphragm in its insertional zone [27].

In a study conducted by Rocha et al., manual diaphragm release technique improved diaphragmatic mobility, 6-minute walking distance, and inspiratory capacity in patients with clinically stable COPD with no history of exacerbations in the past 6 weeks [27].

Abdelaal Ashraf AM et al. studied the effect of diaphragm as well as costal manipulation on functional capacity and pulmonary function in patients with moderate COPD, not involved in previous rehabilitation program at least 4 months prior to the study and had no recent infectious exacerbations for the 2 months preceding the study wherein both techniques (Doming of Diaphragm and Diaphragm Release) were given and it was found that both techniques were effective tools in improving pulmonary function and functional capacity [28].

There was a lack of retrievable data available regarding comparison of Diaphragmatic stretching technique and Manual Diaphragm Release technique on diaphragmatic excursion in patients with COPD. The aim of the study is to compare the effects of Diaphragmatic stretch and Manual Diaphragm Release technique on diaphragmatic excursion in patients with COPD.

## 2. Methodology

### 2.1. Inclusion Criteria

- Patients with stable COPD of both genders who are referred for Physiotherapy by a pulmonologist/physician in KMC Hospitals.
- Patients with mild or moderate COPD according to the GOLD criteria, 2016.GOLD 1: Mild FEV1 ≥ 80% predicted, GOLD 2: Moderate 50% ≤ FEV1 < 80% predicted.

### 2.2. Exclusion Criteria

- Patients with acute exacerbation of COPD.
- Patients with unstable hemodynamic parameters (arterial pressure <100mmHg systolic and <60mmHg for diastolic and mean arterial pressure (MAP) <80mmHg).
- Patients who have undergone recent cardiothoracic or abdominal surgery.
- Patients who have a recent history of chest wall or abdominal trauma; substantial chest wall deformity
- History of psychiatric illness.

## 3. Study Procedure

The study was approved by the Institutional Ethics Committee of Kasturba Medical College Mangalore. Eligible patients were selected based on the inclusion and exclusion criteria. The purpose of study was made clear to each patient and a written informed consent was obtained prior to involving them in the study.

**Figure 1:**
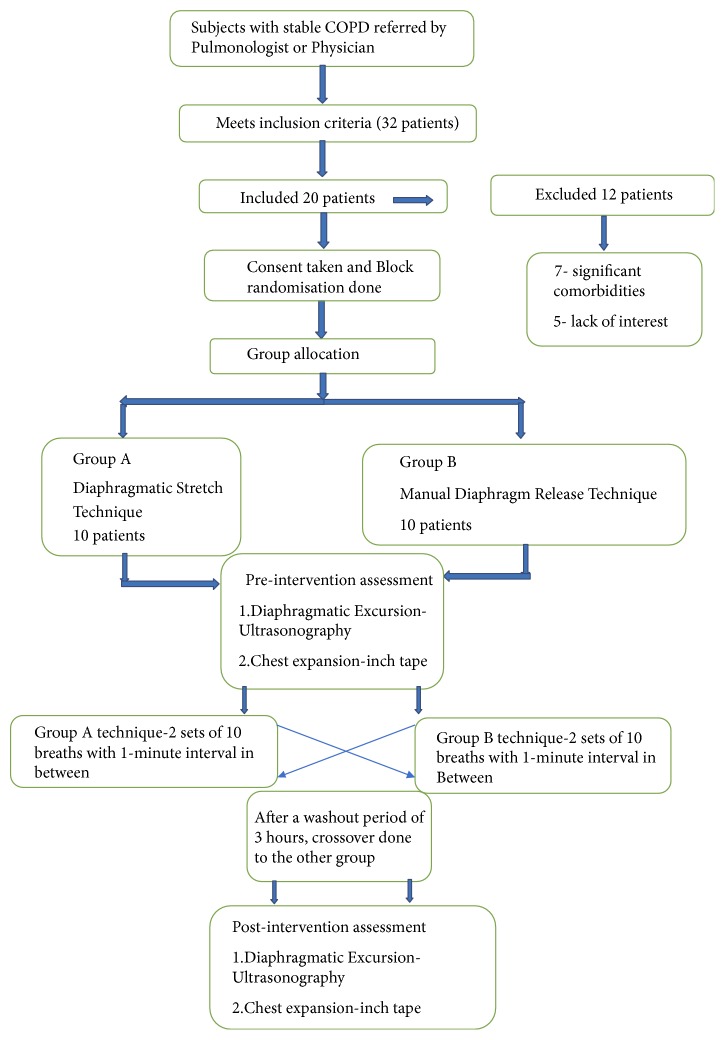
Flow diagram of the study.

The patients were allocated to Group A or Group B by block randomization done by primary investigator. A total of 20 samples were divided into 2 blocks with 10 patients in each. The information about the technique was concealed in a sealed opaque envelope and revealed to the patients only after allocation of groups. After taking the demographic data and baseline values of the outcome measures (diaphragm mobility by ultrasonography performed by an experienced radiologist and chest expansion by inch tape performed by the therapist), Group A subjects underwent the Diaphragmatic Stretch Technique and the Group B subjects underwent the Manual Diaphragm Release Technique. Both the interventions were performed in 2 sets of 10 deep breaths with 1-minute interval between the sets. The two outcome variables were recorded immediately after the intervention. A wash-out period of 3 hours was maintained to neutralize the effect of given intervention. Later the patients of Group A and Group B were crossed over to the other group (Figure 1).

## 4. Methods to Perform the Techniques

### 4.1. Diaphragmatic Stretch Technique

The subjects were asked to sit erect for the intervention. The therapist standing behind the subject pass their hands around the thoracic cage, introducing fingers in the subcostal margins. The subject's trunk was rounded slightly to relax the rectus abdominis. As the subject exhaled, the therapist easing their hands caudally grasped the lower ribs at the subcostal margin. This firm, but gentle, traction was maintained as the patient inhales [22].

### 4.2. Manual Diaphragm Release Technique

The participant was asked to lie supine with relaxed limbs. Positioned at the head of the patient, the therapist made manual contact with the hypothenar region and last three fingers bilaterally to the underside of the seventh to tenth rib costal cartilages. The therapist's forearms were aligned towards the participant's shoulders. In the inspiratory phase, the therapist was gently pulling the points of contact with both hands in the direction of the head and slightly laterally, accompanied the elevation of the ribs.

During exhalation, the therapist's contact was deepened towards the inner costal margin, maintaining resistance. In the subsequent respiratory cycles, the therapist deepened the contact inside the costal margin [27].

## 5. Description of Outcome Measures

### 5.1. Diaphragm Excursion

The patient was asked to sit and diaphragm movements were recorded in the B-Mode. The probe was positioned both in the midclavicular and in the midaxillary lines consecutively, in the subcostal area, so that the ultrasound beam will enter to visualize the bilateral diaphragm perpendicularly.

The procedure began at the end of normal expiration with the subjects instructed to inhale as deeply as possible. A fixed point at the edge of the image on the screen and the diaphragm margin at maximal inspiration and again at maximal expiration served as reference points between which measurements were made, where the averages of three values were taken for both maximal inspiration and maximal expiration [29].

### 5.2. Chest Expansion

The chest expansion was assessed with the patients standing with their hands placed on their head. They were given instructions to “breathe in maximally” and “breathe out maximally.” Chest expansion was measured at two levels. Upper chest expansion at the level of the 4th intercostal space and lower chest expansion at the level of the xiphoid process [30].

## 6. Sample Size Estimation

A pilot study was conducted which involved 5 patients with clinically stable COPD who were crossed over to both groups. Based on the findings of the pilot study, the mean deviation across the crossed over group with respect to midclavicular findings, 90% power, 95% ci, population SD of 0.02, mean value of difference of diaphragmatic stretch technique (0.14), and manual diaphragm release technique (0.08) at midclavicular line and adding 20% nonresponsive error, the total sample was calculated to be 20 subjects using the following formula:

(1) $$\begin{array}{c} \frac{{\left(Z\alpha +Z\beta \right)}^{2}{\sigma_{P}}^{2}}{2{\left(\mu -\mu_{0}-\sigma \right)}^{2}} \end{array}$$

where

  Z*α* – 1.96 for 95% CI.
  Z*β* – 1.34 for 90% power.

### 6.1. Data Analysis

The data were fed into the computer having Statistical Package for Social Science (SPSS) version 11.5. The variables are summarised as mean and standard deviation. The pre and post values for the two techniques were measured using ANOVA. The comparison between the post-intervention values of the two techniques was done using ANOVA and Bonferroni test. A p-value less than 0.05 was considered as statistically significant.

## 7. Results

We selected 32 patients who were diagnosed with clinically stable COPD within the mild or moderate category of GOLD classification. Out of this, 7 patients had to be excluded due to the presence of significant comorbidities (Coronary Artery Disease, Pleural Effusion, Bronchiectasis); 5 patients dropped out of the study due to lack of interest. A total of 20 patients as per the sample size were included on whom both the techniques were performed. Baseline characteristics of the patients such as Age, Gender, COPD category, history of smoking is presented in Table 1. Diaphragmatic Excursion following Diaphragmatic stretch technique, on the Right side there was a difference of 0.29 ±0.21 (p=0.001) in the midclavicular line and 0.25 ±0.20 (p=0.003) in the midaxillary line. On the Left side, there was a difference of 0.24 ±0.24 (p=0.004) in the midclavicular line and 0.35 ±0.25 (p=0.312) in the midaxillary line showed in Table 2.

Diaphragmatic excursion following Manual Diaphragm Release Technique is summarised in Table 3. In Manual Diaphragm Release Technique, on the Right side there was a difference of 0.24 ±0.20(p=0.001) in the midclavicular line and 0.22 ±0.20 (p=0.001) in the midaxillary line. On the Left side, there was a difference of 0.26 ±0.28 (p=0.002) in the midclavicular line and 0.29 ±0.18(p=0.001) in the midaxillary line. Chest expansion values before and after both techniques are summarised in Table 4. After Diaphragmatic Stretch Technique there was a difference of 0.76 ±0.71 (p=0.001) at the level of 4^th^ intercostal space and 0.62 ±0.64 (p=0.001) at the level of xiphoid process. After Manual Diaphragm Release Technique there was a difference of 0.82 ± 0.06 (p=0.002) at the level of 4^th^ intercostal space and 0.72 ±0.88 (p=0.002) at the level of xiphoid process.

Comparison of post values of both techniques in Diaphragmatic Excursion and Chest Expansion is summarised in Table 5. In Diaphragmatic Excursion, difference in postintervention values at the Midclavicular line on the right side was found to be 0.07 ± 0.21 (p= 0.393) and on the left side was found to be -0.04 ± 0.23 (p= 1.00); Difference in post-intervention values at the Midaxillary line on the right side was found to be -0.02 ± 0.26 (p= 1.00) and on the left side was found to be -0.10 ± 0.32 (p= 0.483). In Chest Expansion, difference in the postintervention values at the level of 4^th^ intercostal space was found to be -0.11 ± 0.16 and at the level of xiphoid process was found to be -0.09 ± 0.08

## 8. Discussion

The main purpose of the study was to compare the effects of Diaphragmatic Stretch Technique and Manual Diaphragm Release Technique on Diaphragmatic excursion in COPD. In our study we found that there was a statistically significant difference in the diaphragmatic excursion and chest expansion following both interventions within the groups but there was no significant difference between groups of the two techniques on the two outcome measures.

The Diaphragmatic Stretch Technique was found to have statistically significant within group difference. This can be hypothesised to be due to the acute activation of the muscle spindle caused by muscle stretching, that increases the sensory afferent stimulus, increasing neuromotor response, eventually increasing muscle tension, improving muscle viscoelasticity and consequently decreasing muscle stiffness and increasing thoracic mobility [31–34]. Muscle stretching may stimulate the receptors in the muscle–tendon region i.e. the Golgi tendon organs, thereby causing an inhibitory effect [35, 36].

Noll DR et al reported that one session of manual therapy which included the Redoming of Diaphragm technique, improved the pulmonary function in patients with COPD [11]. Yelvar GDY found that a single session of Manual Therapy which included the Diaphragmatic Release improved the inspiratory muscle strength and pulmonary function in patients with severe COPD [24]. Gonzalez-Alvarez FJ applied the diaphragm stretch technique on healthy subjects and found that there was a significant improvement in ribcage excursion at xiphoid level along with improvement in the posterior chain kinematics [23].

The Manual Diaphragm Release Technique was found to have statistically significant within group difference after may be hypothesised that the technique provided an improvement in the flexibility of the respiratory muscles and the thoracic cavity, as well as an improvement in the length-tension relationship, which allowed a beneficial effect on the performance of respiratory mechanics. This technique may stimulate proprioception and increase the elasticity of adhered fibres, and it acts by eliminating tension in the soft tissues, through low speed movements, which when applied over the area, act on the sensory system through the Golgi tendon organs [37].

However, there is little research that gives scientific support to the effects of such techniques. In the literature there is a deficiency of studies on the action of stretching of the respiratory muscles, affirming to us that this probably occurs because it is a muscular group of complex functioning and, perhaps for this reason, does not present specific techniques [37].

Rocha et al, performed the Manual Diaphragm Release Technique in stable COPD patients and found an improvement in diaphragm mobility [27]. Abdaleel Ashraf AM et al. found that application Diaphragmatic Release technique and Redoming of the Diaphragm technique significantly increased FVC, FEV1 and 6MWT [28]. Braga DKAP et al found that the “diaphragm lift” and double diaphragm brought about an improvement in the maximum expiratory pressure, all the coefficients of the cirtometry and mobility of the thoracic cavity [37].

In our study, we found the between groups values to be statistically nonsignificant. This may be due to a small sample size, which must have hindered with the comparison of both the techniques. It can also be attributed to the fact that the number of repetitions done for both the techniques was not sufficient enough to compare between them.

The limitations of the study are that larger sections of the COPD population should be included. We have measured the immediate effects of the techniques on the diaphragmatic excursion. The skill and expertise of the therapist in performing the techniques are a subjective limitation of the study. Further studies may be done for a longer duration using both the techniques in patients belonging to different COPD subgroups.

## 9. Conclusion

The Diaphragmatic Stretch Technique and Manual Diaphragm Release Technique can be safely recommended for patients with clinically stable COPD to improve Diaphragmatic Excursion and Chest Expansion.

## Figures and Tables

**Table 1:** Demographic data of participants.

**Variable**		**Frequency**
Age (66.85 ± 8.37)		20

Gender	male	12
	Female	8

Smoking history	yes	9
	No	11

COPD category	mild	11
	Moderate	9

**Table 2:** Comparison of Diaphragmatic Excursion before and after Diaphragmatic Stretch Technique.

	**Right Side (in cms)**				**Left Side (in cms)**			
**Midclavicular Line**	**Pre**	**Post**	**Difference**	**P <0.05**	**Pre**	**Post**	**Difference**	**P<0.05**
	2.56 ± 0.56	2.86 ± 0.59	0.29 ± 0.21	0.001*∗∗*	2.57 ± 0.54	2.79 ± 0.52	0.24 ± 0.24	0.004*∗∗*

**Midaxillary Line**	**Pre**	**Post**	**Difference**	**P <0.05**	**Pre**	**Post**	**Difference**	**P<0.05**
	2.74 ± 0.63	2.95 ± 0.70	0.25 ± 0.20	0.003*∗∗*	2.69 ± 0.63	2.85 ± 0.6	0.35 ± 0.25	0.312

Note: *∗∗* is highly significant.

**Table 3:** Comparison of Diaphragmatic Excursion before and after the Manual Diaphragm Release Technique.

	**Right (in cms)**				**Left (in cms)**			
**Midclavicular Line**	**Pre**	**Post**	**Difference**	**p<0.05**	**Pre**	**Post**	**Difference**	**p<0.05**
	2.56 ± 0.56	2.78 ± 0.52	0.24 ± 0.20	0.001*∗∗*	2.57 ± 0.54	2.84 ± 0.59	0.26 ± 0.28	0.002*∗∗*

**Midaxillary Line**	**Pre**	**Post**	**Difference**	**p<0.05**	**Pre**	**Post**	**Difference**	**p<0.05**
	2.74 ± 0.63	2.98 ± 0.62	0.22 ± 0.20	0.001*∗∗*	2.69 ± 0.63	2.95 ± 0.55	0.29 ± 0.18	0.001*∗∗*

Note: *∗∗* is highly significant.

**Table 4:** Comparison of Chest Expansion before and after Diaphragmatic Stretch Technique and Manual Diaphragm Release Technique.

	4^**th**^** intercostal space (in inches)**				**Xiphoid process (in inches)**			
**Diaphragmatic Stretch **	**Pre**	**Post**	**Difference**	**p<0.05**	**Pre**	**Post**	**Difference**	**p<0.05**
	34.98 ± 2.95	35.69 ± 2.85	0.76 ± 0.71	0.001	36.10 ± 3.22	36.73 ± 3.26	0.62 ± 0.64	0.001

**Manual Diaphragm Release Technique**	**Pre**	**Post**	**Difference**	**p<0.05**	**Pre**	**Post**	**Difference**	**p<0.05**
	34.98 ± 2.95	35.80 ± 3.01	0.82 ± 0.06	0.002	36.10 ± 3.22	36.82 ± 3.34	0.72 ± 0.88	0.002

**Table 5:** Comparison of post intervention values of Diaphragmatic Excursion and Chest Expansion between groups.

**Diaphragmatic Excursion (in cms)**					

Technique		Midclavicular line		Midaxillary line	
		Right side	Left side	Right side	Left side

Diaphragmatic stretch technique		2.86 ± 0.59	2.79 ± 0.52	2.95 ± 0.70	2.85 ± 0.6

Manual diaphragm release technique		2.78 ± 0.52	2.84 ± 0.59	2.95 ± 0.70	2.95 ± 0.55

Difference	Mean ± SD	0.07 ± 0.21	-0.04 ± 0.23	-0.02 ± 0.26	-0.10 ± 0.32
	sig. (p< 0.05)	0.393	1.00	1.00	0.483

**Chest expansion (in inches)**					

Technique		4^th^ intercostal space		Xiphoid process	

Diaphragmatic stretch technique		35.69 ± 2.85		36.73 ± 3.26	

Manual diaphragm release technique		35.80 ±3.01		36.82 ± 3.34	

Difference	Mean ± SD	-0.11 ± 0.16		-0.09 ± 0.08	
	sig. (p< 0.05)	0.713		0.737	

## Data Availability

The data used to support the findings of this study are available from the corresponding author upon request.
